# Downregulation of the phosphatase JKAP/DUSP22 in T cells as a potential new biomarker of systemic lupus erythematosus nephritis

**DOI:** 10.18632/oncotarget.11419

**Published:** 2016-08-19

**Authors:** Huai-Chia Chuang, Yi-Ming Chen, Wei-Ting Hung, Ju-Pi Li, Der-Yuan Chen, Joung-Liang Lan, Tse-Hua Tan

**Affiliations:** ^1^ Immunology Research Center, National Health Research Institutes, Zhunan, Taiwan; ^2^ Division of Allergy, Immunology, and Rheumatology, Taichung Veterans General Hospital, Taichung, Taiwan; ^3^ Faculty of Medicine, National Yang-Ming University, Taipei, Taiwan; ^4^ Rong Hsing Research Center for Translational Medicine, National Chung Hsing University, Taichung, Taiwan; ^5^ School of Medicine, China Medical University Hospital, Taichung, Taiwan; ^6^ Division of Rheumatology and Immunology, China Medical University Hospital, Taichung, Taiwan; ^7^ Institute of Biotechnology, National Tsing Hua University, Hsinchu, Taiwan; ^8^ Department of Pathology & Immunology, Baylor College of Medicine, Houston, Texas, USA

**Keywords:** JKAP, DUSP22, SLE, nephritis, T cells, Immunology and Microbiology Section, Immune response, Immunity

## Abstract

Systemic lupus erythematosus (SLE) is a complex autoimmune disease that is characterized by systemic inflammation and multiple organ failures. Dysregulation of T cells plays a critical role in SLE pathogenesis. Our previous study indicates that JKAP (also named DUSP22) inhibits T-cell activation and that JKAP knockout mice develop spontaneous autoimmunity; therefore, we investigated whether JKAP downregulation is involved in SLE patients. JKAP protein levels in purified T cells were examined by immunoblotting using blood samples from 43 SLE patients and 32 healthy controls. SLE patients showed significantly decreased JKAP protein levels in peripheral blood T cells compared to healthy controls. JKAP protein levels in peripheral blood T cells were inversely correlated with SLE disease activity index (SLEDAI) and anti-dsDNA antibody levels. JKAP downregulation in T cells was highly correlated with daily urinary protein amounts and with poor renal outcome in lupus nephritis patients. Notably, the diagnostic power of JKAP downregulation in T cells for active lupus nephritis was higher than those of serum anti-dsDNA antibody, C3, and C4 levels. Moreover, T-cell-specific transgenic mice expressing a dominant-negative JKAP mutant developed spontaneous autoimmune nephritis. Furthermore, JKAP-deficient T cells overproduced complement components, soluble ICAM-1, and soluble VCAM-1 in the kidney; these cytokines have been reported to be involved in lupus nephritis. Taken together, JKAP downregulation in T cells is a novel diagnostic and prognostic biomarker for SLE nephritis.

## INTRODUCTION

Systemic lupus erythematosus (SLE) is an autoimmune disease that is characterized by pathogenic autoantibody production, systemic inflammation, and multiple organ failures [1]. Lupus nephritis, a debilitating kidney disease, is a severe manifestation of SLE [2]. Lupus nephritis often occurs within the first two years after SLE diagnosis [3]. Up to 60% of SLE patients will develop lupus nephritis, which is associated with morbidity and mortality [4]. Early detection and adequate therapy are associated with better outcomes in lupus nephritis. Diagnosis of lupus nephritis is usually based on assessment of renal function, quantification of daily urinary protein, and microscopic examination of urine. The conventional biomarkers for SLE, including complement components 3 and 4 (C3, C4) and anti-double-stranded DNA antibodies (ANA), have low sensitivity (49-79%) and specificity (51-74%) for concurrent renal flare [3]. To date, none of above parameters is predictive of activity and severity of lupus nephritis evaluated by the invasive renal biopsy. Thus, it is pivotal to identify novel diagnostic biomarkers for SLE nephritis.

Dysregulation of the immune system, including abnormal T-cell, B-cell, and dendritic-cell responses, participates in the pathogenesis of SLE [5, 6]. In particular, SLE T cells have dysregulated signaling pathways and participate in the progression of SLE and the development of organ damage [7]. Several dual-specificity phosphatases (DUSPs) negatively regulate T-cell activation and signaling [8–12]. JNK pathway-associated phosphatase (JKAP, also named DUSP22) specifically activates the kinase JNK [13]. JKAP acts as a tyrosine phosphatase to dephosphorylate and inactivate focal adhesion kinase, leading to the suppression of cell motility [14]. JKAP negatively regulates T-cell receptor (TCR) signaling by dephosphorylating and inhibiting the tyrosine kinase Lck; JKAP knockout (KO) mice spontaneously develop autoimmune diseases [10]. In this report, we studied whether JKAP downregulation in T cells is associated with human SLE.

## RESULTS

### JKAP protein levels is downregulated in peripheral blood T cells from SLE patients

To examine the clinical relevance of JKAP in human systemic lupus erythematosus (SLE) patients, we examined peripheral blood samples freshly isolated from 43 SLE patients and 32 healthy controls. A total of 43 SLE patients were enrolled between September 15, 2010 and October 21, 2015. The baseline demographics of the human subjects are listed in Table 1. The specimens were from 43 enrolled SLE patients with an average age of 34.8 ± 12.6 (mean ± SD) years compared to 32 enrolled healthy controls with an average age of 38.7 ± 15.0. There were 40 females (93.0%) and 25 females (80.6%) in the SLE patients and the healthy controls, respectively. The SLE disease activity index (SLEDAI) of enrolled lupus patients was 7.5 ± 6.3. Among these 43 SLE patients, 12 (27.9%) patients developed active renal flares, 2 (4.6%) developed CNS manifestation, 8 (18.6%) developed active arthritis, 4 (9.3%) developed leukopenia or thrombocytopenia, 22 (51.2%) developed mucocutaneous flares, 23 (53.5%) displayed high anti-dsDNA antibody levels or low C3/C4 complement levels. Forty patients (93.0%) were treated with hydroxychloroquine (HCQ), 38 (88.4%) with prednisone, 24 (55.8%) with azathioprine (AZA), 5 (11.6%) with mycophenolate mofetil (MMF), and 3 (7.0%) with cyclophosphamide (CYC).

**Table 1:** Baseline demographic and disease characteristics of SLE patients and healthy controls

Characteristic	SLE (*n* = 43)	HC (*n* = 32)
Age at study entry (years)	34.8 ± 12.6	38.7 ± 15.0
No. of female (%)	40 (93.0)	25 (80.6)
Duration (years)	9.0 ± 7.4	N/A
SLEDAI	7.5 ± 6.3	N/A
WBC (cells/mm^3^)	5806 ± 2149	N/A
HgB (g/dl)	12.4 ± 2.1	N/A
Platelet (10^3^ cells/mm^3^)	246.3 ± 81.1	N/A
Serum creatinine (mg/dl)	0.73 ± 0.24	N/A
Anti-dsDNA antibody (WHO units/ml)	220.3 ± 178.9	N/A
Serum C3 (mg/dl)	81.9 ± 25.7	N/A
Serum C4 (mg/dl)	17.4 ± 7.7	N/A
Daily urinary protein (g/day)	1.2 ± 3.1	N/A

Abbreviations: SLE, systemic lupus erythematosus; HC, healthy controls; SLEDAI, SLE disease activity index; WBC, white blood cell; HgB, hemoglobin; dsDNA, double-stranded DNA; WHO, World Health Organization; C3 and C4, complement component 3 and component 4; N/A, non-applicable.

Plus-minus values are means ± SD. Demographic characteristics were determined only at the beginning of SLE diagnosis. Durations refer to the length of the disease time after SLE diagnosis.

**Figure 1:**
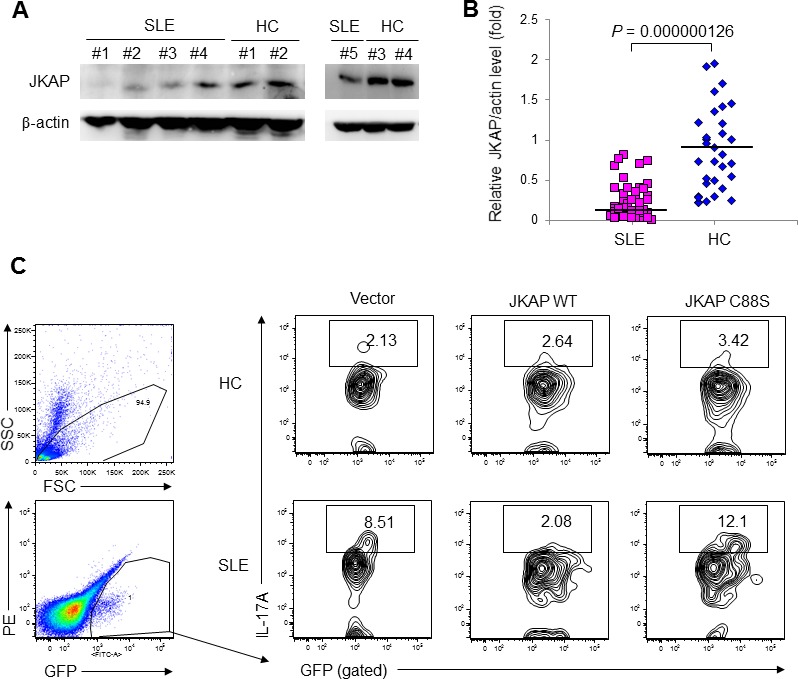
JKAP levels are reduced in T cells from human SLE patients **A.** Immunoblotting of JKAP levels in purified peripheral blood T cells from five representative SLE patients and four representative healthy controls (HC). **B.** Densitometry analyses of the immunoblotting data from 43 SLE patients and 32 HC. Relative fold changes were normalized to actin. Each symbol represents an individual subject. Two-tailed Student's *t*-test, *P* = 0.000000126. **C.** Flow cytometry analyses of IL-17A-positive cells from HC and SLE T cells transfected with empty vector encoding GFP alone, GFP-tagged JKAP wild-type (WT), or GFP-tagged JKAP C88S phosphatase-dead mutant.

We found that JKAP protein levels were significantly reduced in purified peripheral blood T cells of human patients with SLE (JKAP/actin = 0.24 ± 0.05 fold) compared to those of healthy controls (JKAP/actin = 0.87 ± 0.26 fold) as determined by immunoblotting analyses (Figure 1A and 1B; *P* = 1.26×10^-7^). The mRNA levels of JKAP were not significantly changed in purified peripheral blood T cells from SLE patients (Supplementary Figure S1), suggesting that JKAP downregulation in SLE peripheral blood T cells may be due to enhanced degradation or decreased synthesis of the JKAP protein. JKAP knockout mice display spontaneous systemic inflammation and increased serum IL-17A levels [10]. To demonstrate that JKAP downregulation in T cells led to T-cell activation in SLE patients, GFP-tagged JKAP plasmids were transfected into purified peripheral T cells from SLE patients. The percentage of IL-17A-producing T cells from SLE patients was higher than that from healthy controls; the increased percentage was abolished by transfected JKAP but not JKAP phosphatase-dead mutant (Figure 1C). The data demonstrate that JKAP is downregulated in peripheral blood T cells from SLE patients and suggest that reduced JKAP protein levels may contribute to the dysregulation of lupus T cells.

### JKAP protein levels in T cells are significantly decreased in active lupus nephritis patients

As the dysfunction of lupus T cells may result in the pathogenesis of SLE, we studied whether JKAP protein levels in peripheral blood T cells are associated with the clinical symptoms of SLE patients. We found that downregulation of JKAP protein levels in peripheral blood T cells were significantly correlated with fever (*P* = 0.022) and nephritis (*P* = 0.001) but not with rash, arthritis, cutaneous vasculitis, oral ulcer, serositis or CNS involvement in SLE patients (Table 2). In particular, JKAP protein levels in peripheral blood T cells from active lupus nephritis patients (JKAP/actin = 0.09 ± 0.14 fold, *n* = 12; *P* = 0.003) were more reduced than those from inactive lupus nephritis patients (JKAP/actin = 0.34 ± 0.27 fold, *n* = 9) and non-nephritis patients (JKAP/actin = 0.34 ± 0.24 fold, *n* = 22; Table 2). As expected, SLEDAI or daily urinary protein levels were significantly increased and serum C3 levels were significantly decreased in active lupus nephritis patients compared to those in inactive lupus nephritis patients (Table 3). Similar to the above nephritis-associated clinical parameters, JKAP protein levels in T cells were also significantly decreased in active lupus nephritis patients (Table 3). The data suggest that downregulation of JKAP in peripheral blood T cells may be associated with lupus nephritis among SLE patients.

**Table 2:** Comparison of JKAP protein levels in T cells with various symptoms among SLE patients

Symptom	*n*	JKAP/actin (fold)	*P*
Fever^†^			
Yes	9	0.13 ± 0.16	0.022^*^
No	34	0.31 ± 0.25	
Rash^†^			
Yes	22	0.27 ± 0.27	0.551
No	21	0.27 ± 0.22	
Arthritis^†^			
Yes	8	0.25 ± 0.26	0.708
No	35	0.28 ± 0.25	
Cutaneous vasculitis^†^			
Yes	10	0.20 ± 0.19	0.357
No	33	0.29 ± 0.26	
Oral ulcer^†^			
Yes	2	0.14 ± 0.02	0.604
No	41	0.28 ± 0.25	
Serositis^†^			
Yes	5	0.14 ± 0.17	0.103
No	38	0.29 ± 0.25	
CNS^†^			
Yes	2	0.04 ± 0.02	0.066
No	41	0.28 ± 0.25	
Nephritis^¶^^#^^§^			
Active	12	0.09 ± 0.14	0.001^*^^¶^
Inactive	9	0.34 ± 0.27	0.003^*^^#^
No	22	0.34 ± 0.24	0.001^*^^§^

Plus-minus values are means ± SD.

† *P* values were calculated with the *Mann*–Whitney *U test.*

¶ *P* values were calculated with the Kruskal–Wallis test.

#, § *P* values of^#^Active vs. Inactive or ^§^Active vs. No nephritis was calculated with the post-hoc analysis.

* *P*-value < 0.05, statistical significance.

The effects of immunosuppressive agents using for SLE treatment have been shown to inhibit T-cell activation and induce lymphocyte death [15]. To rule out the possibility that JKAP downregulation in SLE T cells is due to any indirect effects of therapeutic agents, we studied the relationships between JKAP protein levels and medical treatments. JKAP protein levels in T cells were not significantly different among SLE patients between the groups treated with prednisone, HCQ, CYC, AZA, or MMF and the groups that were not treated with any of those (data not shown). Moreover, JKAP protein levels in peripheral blood T cells were not significantly correlated with the prednisone dosages in SLE patients (data not shown). The data showed that the treatment of immunosuppressive agents does not contribute to JKAP downregulation in SLE T cells.

**Table 3:** Clinical characteristics of inactive and active lupus nephritis (LN)

	Inactive LN (*n* = 9)	Active LN (*n* = 12)	^†^*P*
JKAP/actin (fold)	0.34 ± 0.27	0.09 ± 0.14	0.003^*^
SLEDAI	4.0 ± 4.6	12.8 ± 7.6	0.007^*^
WBC (cells/mm^3^)	5343 ± 2094	5930 ± 2162	0.651
HgB (g/dl)	12.6 ± 2.3	11.8 ± 2.7	0.422
Platelet (10^3^ cells/mm^3^)	239.6 ± 99.8	247.9 ± 108.9	0.554
Serum creatinine (mg/dl)	0.74 ± 0.16	0.83 ± 0.28	0.554
Anti-dsDNA antibody (WHO units/ml)	117.6 ± 112.5	298.0 ± 216.4	0.058
Serum C3 (mg/dl)	92.9 ± 21.3	65.1 ± 30.8	0.034^*^
Serum C4 (mg/dl)	17.8 ± 6.4	14.0 ± 10.6	0.193
Daily urinary protein (g/day)	0.15 ± 0.04	2.72 ± 3.96	0.001^*^

Abbreviations: SLEDAI, SLE disease activity index; WBC, white blood cell; HgB, *hemoglobin*; dsDNA, double-stranded DNA; WHO, World Health Organization; C3 and C4, complement component 3 and component 4.

Plus-minus values are means ± SD.

† *P* values were calculated with the use of the *Mann*–Whitney *U test*.

* *P*-value < 0.05, statistical significance.

### JKAP downregulation in T cells correlates with nephritis and poor renal outcome in SLE patients

**Figure 2:**
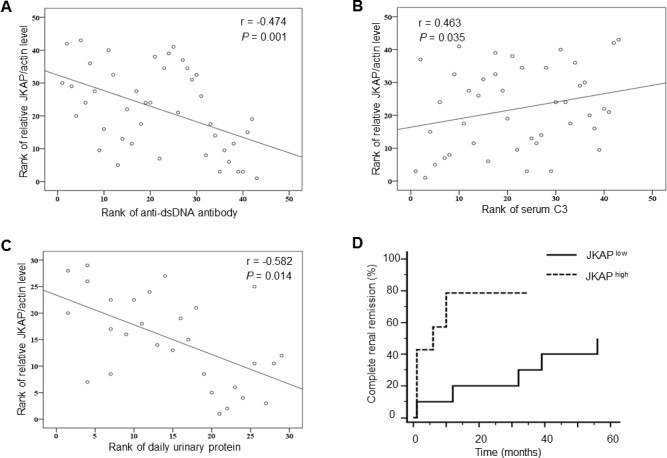
JKAP downregulation correlates with nephritis and poor renal outcome **A.**-**C.** Spearman's rank correlation of JKAP protein levels with anti-dsDNA antibody (A), serum C3 levels (B), and daily urinary protein levels (C). **D.** Comparisons of complete renal remission rates between lupus nephritis patients with high JKAP levels and those with low JKAP levels during 56 months by Kaplan-Meier method. *P* = 0.0218.

We further examined the association between JKAP protein levels in peripheral blood T cells and the clinical manifestations in SLE. JKAP protein levels were positively correlated with white blood cell numbers (WBC; *r* = 0.384, *n* = 43, *P* = 0.011), while JKAP protein levels were inversely correlated with SLEDAI (*r* = −0.398, *n* = 43, *P* = 0.008) and anti-dsDNA antibody levels (*r* = −0.474, *n* = 43, *P* = 0.001; Table 4 and Figure 2A). Serum levels of complements are widely used to monitor nephritis activity of SLE nephritis patients. Thus, we further analyzed the correlation between JKAP levels in T cells and serum complement levels using lupus nephritis patients; JKAP protein levels in T cells were significantly correlated with serum C3 levels in lupus nephritis patients (*r* = 0.463, *n* = 21, *P* = 0.035; Table 4 and Figure 2B). Furthermore, urinary protein amounts are useful for characterizing renal disease activity, such as lupus nephritis; urinary protein excretion (daily urinary protein) was measured using 24-hour urine collection [16]. There were 21 daily urinary protein data from individuals. Notably, JKAP downregulation was highly correlated with daily urinary protein amounts (*r* = −0.582, *n* = 21, *P* = 0.014; Table 4 and Figure 2C). In addition, renal biopsy is the mainstay for the clinical diagnosis of lupus nephritis [16]. Eight SLE patients in this cohort received renal biopsy. Three of them had class III lupus nephritis; three had class IV; one had class V and one had mixed III & V. Most of them had active lupus nephritis with pathological activity scores ranging from 3 to 10. In these renal biopsy specimens, only minimal chronicity scores (< 2) were observed. JKAP protein levels in T cells were much lower in Class IV lupus nephritis patients (JKAP/actin = 0.02 ± 0.01 fold) than those in the other five patients (JKAP/actin = 0.13 ± 0.13 fold). Two of them had active urinary sediments, and their relative JKAP to actin levels were very low (JKAP/actin = 0.02 fold and 0.11 fold, respectively).

**Table 4:** Correlations between JKAP protein levels in T cells and clinical manifestations in SLE patients

Clinical manifestations	*n*	*r*	^†^*P*
SLEDAI	43	−0.398^*^	0.008^*^
Renal SLEDAI	43	−0.473^*^	0.001^*^
WBC	43	0.384^*^	0.011^*^
HgB	43	0.215	0.167
Platelet	43	0.080	0.612
Serum creatinine	43	−0.099	0.528
Anti-dsDNA antibody	43	−0.474^*^	0.001^*^
Serum C3	21	0.463^*^	0.035^*^
Serum C4	21	0.242	0.290
Daily urinary protein	21	−0.582^*^	0.014^*^

Abbreviations: SLEDAI, SLE disease activity index; WBC, white blood cell; HgB, *hemoglobin*; dsDNA, double-stranded DNA; C3 and C4, complement component 3 and component 4.

† *P* values were calculated with the Spearman's rank correlation test.

* *P* value < 0.05, statistical significance.

**Figure 3:**
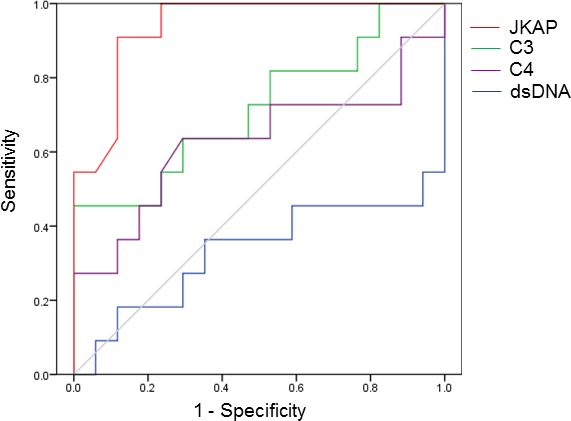
The diagnostic power of JKAP downregulation is higher than those of existing biomarkers Receiver operating characteristic (ROC) curves of the JKAP protein, C3, C4, and anti-dsDNA antibody levels for detection of active lupus nephritis.

To study whether the JKAP protein in T cells is a useful diagnostic biomarker for active lupus nephritis, the diagnostic utility of the JKAP protein and existing diagnostic biomarkers were analyzed by multivariate logistic regression and ROC curve analyses. JKAP protein level in T cells was the only variate that significantly correlated with the active lupus nephritis after comparison with serum levels of dsDNA, C3, and C4 (*n* = 43, odds ratio = 0.000028, *P* = 0.029; Table 5). This result indicates that JKAP downregulation is an independent biomarker for active lupus nephritis. Next, the diagnostic power for active lupus nephritis was analyzed by ROC curve analysis using the JKAP protein, C3, C4, and anti-dsDNA antibody levels of the SLE patients who may have nephritis. Using a cutoff value of 0.113 JKAP/actin, JKAP protein level in T cells had a sensitivity of 90.9% and a specificity of 88.2% for identifying SLE patients with active nephritis (Figure 3). Notably, the area under the curve (AUC) value of JKAP protein level (0.939, *P* < 0.001) was significantly higher than that of C3 level (0.717, *P* = 0.057), C4 level (0.628, *P* = 0.259), or anti-dsDNA antibody level (0.332, *P* = 0.138). These data suggest that JKAP downregulation in T cells is a useful diagnostic biomarker for active lupus nephritis.

**Table 5:** Multivariate logistic regression analysis of active lupus nephritis with the JKAP protein and other existing biomarkers

Parameters	Odds Ratio	95% CI	*P*
JKAP/actin (fold)	2.8 × 10^-5^	5.4 × 10^-9^−0.14	0.029^*^
Serum C3 (mg/dl)	0.938	0.87–1.01	0.910
Serum C4 (mg/dl)	1.028	0.87–1.21	0.143
Anti-dsDNA antibody (WHO units/ml)	0.997	0.99–1.00	0.109

Abbreviations: dsDNA, anti-double-stranded DNA antibody; WHO, World Health Organization; C3 and C4, complement component 3 and component 4.

CI, confidence interval

* *P*-value < 0.05, statistical significance.

To test whether JKAP is a useful biomarker in longitudinal observational study, we compared the complete renal remission rates during 56 months of SLE nephritis patients who had high JKAP expression levels with those who had low JKAP expression levels. Patients with high JKAP expression levels showed significantly higher complete renal remission rates than those with low JKAP expression levels (*P* = 0.0218, Figure 2D). These data suggest that JKAP downregulation may be a useful diagnostic biomarker for SLE, especially for active lupus nephritis.

Because JKAP dephosphorylates and inactivates Lck in T cells [10], we studied whether JKAP downregulation results in Lck activation in SLE T cells. Indeed, the SLE T cells with JKAP downregulation (< 0.2 fold) displayed higher levels of phospho-Y394-Lck (SLE #6 and #10) than healthy T cells; however, Lck phosphorylation was also enhanced in the SLE T cells without JKAP downregulation (SLE #7 and #11) (Supplementary Figure S2A). The percentages of phospho-Y394-Lck-positive T cells in peripheral blood from SLE patients (12.68 ± 1.94 %) were higher than those from healthy controls (4.34 ± 0.82 %) examined using flow cytometry (*P* = 0.0031789, *n* = 32; Supplementary Figure S2B, C). Although the SLE patients with greatly decreased JKAP levels in T cells (< 0.2 fold) all exhibited the increased percentages of phospho-Lck-positive T cells (> 4.34%) compared to healthy controls, other SLE patients with normal JKAP levels in T cells also had increased population of phospho-Lck-positive T cells (Supplementary Figure S3). The data indicate that JKAP downregulation and other unknown factors contribute to Lck hyperactivation. Moreover, the percentage of phospho-Lck was not correlated with SLEDAI (*r* = 0.176, *P* = 0.547), nephritis (Kruskal-Wallis test, *P* = 0.089), or other clinical parameters (Supplementary Table S1), suggesting that Lck hyperactivation is a secondary event. These results also indicate that Lck activation is not useful to be used as a biomarker for SLE or SLE nephritis.

### Inflammation and autoimmunity in Lck-JKAP-C88S transgenic mice

**Figure 4:**
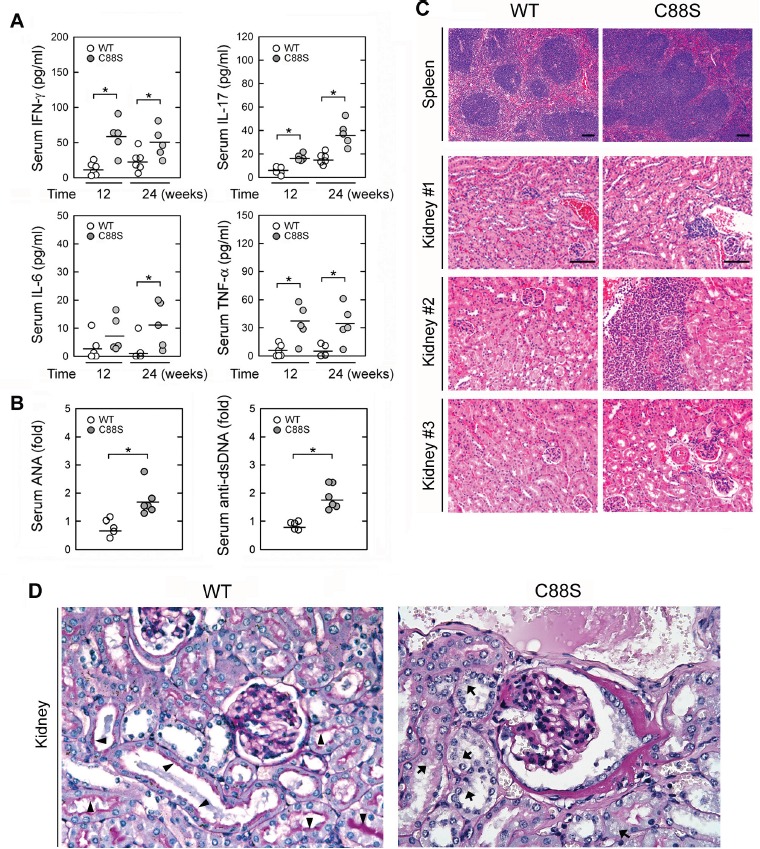
Lck-JKAP-C88S transgenic mice develop spontaneous inflammation and autoimmunity **A.** ELISAs of serum IFN-γ, IL-17, IL-6, and TNF-α levels in 12- and 24-week-old Lck-JKAP-C88S transgenic mice and age-matched wild-type mice. **B.** ELISAs of serum anti-nuclear antibody (ANA) and anti-dsDNA levels in 24-week-old Lck-JKAP-C88S transgenic mice and age-matched wild-type mice. The ANA and anti-dsDNA levels are presented relative to the value from one of control wild-type mice. **C.** Photomicrographs of the spleen and kidney tissues from 24-week-old Lck-JKAP-C88S transgenic mice and age-matched wild-type mice stained with hematoxylin-eosin. Scale bar: 100 μm. **D.** Representative micrographs of periodic acid-Schiff (PAS)-stained kidney sections from 52-week-old Lck-JKAP-C88S transgenic mice and their wild-type littermates. Arrows, brush borders. Arrowheads, protein droplets. At least four mice were analyzed for each group. Two-tailed Student's *t*-test, *, *P* < 0.05. Bars show the mean ± SEM. WT, wild-type; C88S, Lck-JKAP-C88S transgenic mice.

Our previous publication reported that whole-body JKAP knockout mice spontaneously develop autoimmune diseases with nephritis [10]. To demonstrate that loss of JKAP in T cells alone can cause autoimmune diseases and nephritis *in vivo* using an animal model, we generated T-cell specific Lck-JKAP-C88S transgenic mice, which expressed dominant-negative JKAP-C88S mutant proteins specifically in splenic T cells (Supplementary Figure S4A-C). The T-cell lineage in the thymus Lck-JKAP-C88S transgenic mice was normal compared to wild-type mice (Supplementary Figure S4D). Serum levels of proinflammatory cytokines, including IFN-γ, IL-17, IL-6, and TNF-α, were increased in 12- and 24-week-old Lck-JKAP-C88S transgenic mice compared to those of age-matched wild-type mice (Figure 4A). Furthermore, higher levels of anti-clear antibodies (ANA) and anti-dsDNA antibodies were detected in the sera from 24-week-old Lck-JKAP-C88S transgenic mice (Figure 4B). These data demonstrate that overexpression of JKAP-C88S mutant proteins in T cells promotes T-cell hyperactivation, leading to spontaneous inflammation in mice. Consistently, the 24-week-old Lck-JKAP-C88S transgenic mice (7/7, 100%) showed the expansion of white pulps in spleens and the infiltration of lymphocytes in kidney renal tubules, liver tissues, and lung tissues (Figure 4C and Supplementary Figure S5). Both male and female Lck-JKAP-C88S transgenic mice displayed similar phenotypes (data not shown). Conversely, the age-matched wild-type mice showed no obvious abnormalities in pathology (Figure 4C and Supplementary Figure S5). Moreover, the infiltration of T cells into the kidneys was enhanced in Lck-JKAP-C88S transgenic mice (Supplementary Figure S6). The mesangial cell proliferation was also enhanced in the kidney of Lck-JKAP-C88S transgenic mice (Figure 4D). The swollen tubular cells of Lck-JKAP-C88S transgenic mice also showed accumulation of protein droplets and loss of brush borders (Figure 4D), which are symptoms of proteinuria. These results further demonstrate that JKAP deficiency-mediated T-cell hyperactivation in mice could develop autoimmune disorders and nephritis.

**Figure 5:**
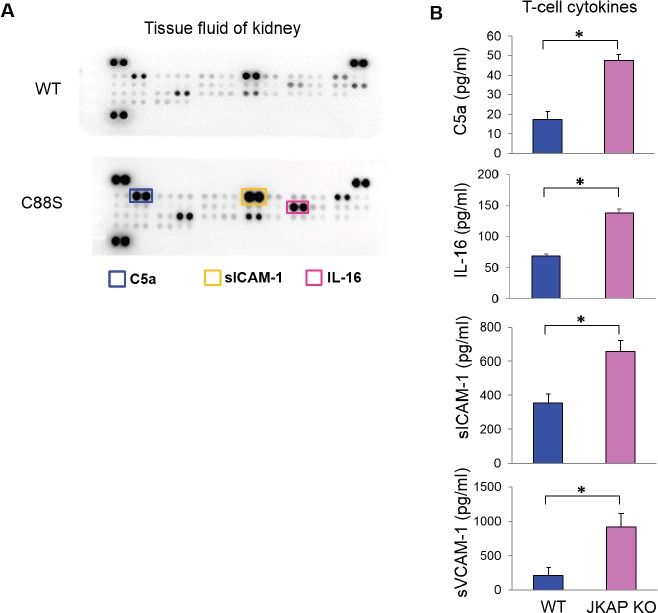
JKAP-deficient T cells produce more complement component C5a and cytokines **A.** Kidney tissue fluids from 6-month-old wild-type and Lck-JKAP-C88S transgenic mice were subjected to the cytokine array analyses. The cytokines whose levels were increased in Lck-JKAP-C88S transgenic mice are labeled by colored rectangles. WT, wild-type; C88S, Lck-JKAP-C88S transgenic mice. **B.** Primary splenic T cells purified from 12-week-old wild-type mice and JKAP knockout (KO) mice were cultured for 3 days without any stimulation. The C5a, IL-16, soluble ICAM-1, and soluble VCAM-1 levels in the supernatants were determined by ELISA assays. Two-tailed Student's *t*-test, *, *P* < 0.05. Bars show the mean ± SEM.

Next, we examined which T-cell cytokines are enhanced in the kidneys of JKAP-deficient mice using cytokine array analyses. We found that the levels of C5a, soluble ICAM-1, and IL-16 were enhanced in the kidney fluids of Lck-JKAP-C88S transgenic mice compared to those of wild-type mice (Figure 5A). In contrast, the urine levels of C5a, soluble ICAM-1, and IL-16 detected by ELISA assays were not significantly increased in Lck-JKAP-C88S transgenic mice compared to those of wild-type mice (Supplementary Figure S7). In order to study whether JKAP downregulation in T cells results in the enhancement of these secreted molecules, splenic T cells were isolated from JKAP knockout mice10 and subjected to ELISA assays. JKAP knockout T cells produced more C5a, IL-16, soluble ICAM-1, and soluble VCAM-1 than those of wild-type T cells (Figure 5B). These data are consistent with previous reports that complement components, soluble ICAM-1, IL-16, and soluble VCAM-1 are potential biomarkers of lupus nephritis [17, 18]. Taken together, JKAP downregulation in T cells results in overproduction of inflammatory cytokines and immune complements, which may contribute to nephritis.

## DISCUSSION AND CONCLUSIONS

Lupus nephritis is a serious and frequent symptom in SLE. The pathogenesis of *lupus nephritis* involves multiple mechanisms, including the activation of complements, deposition of autoantibodies in the glomerulus, as well as the production of extracellular matrix proteins, proinflammatory cytokines, and chemokines [19]. T cells are major inflammatory cells within the inflamed kidneys in human SLE patients and mouse SLE models [20, 21]. Adoptively transferred T cells can cause renal injury in SCID mice [22]. T-cell signaling and gene transcription abnormalities also participate in the pathogenesis of SLE [7]. Our recent study demonstrates that aged JKAP knockout mice spontaneously develop systemic inflammation and autoimmunity, including exacerbated renal damage; mice containing adoptively transferred JKAP knockout T cells are more susceptible to experimental autoimmune encephalomyelitis [10]. Consistently, this report shows that JKAP protein levels were significantly decreased in T cells of human SLE patients, particular in SLE patients with nephritis. Here we also report that T-cell specific dominant-negative JKAP mutant transgenic mice spontaneously developed autoimmune nephritis and autoantibodies. Moreover, JKAP knockout T cells displayed overproduction of C5a, soluble ICAM-1, and soluble VCAM-1, which are also overexpressed in the kidneys of T-cell specific dominant-negative JKAP mutant transgenic mice. Immune complements play important roles in the damage of glomerulus [23] and in the activation of effector T cells [24]. Soluble ICAM-1 and VCAM-1 have been reported to be chemotactic for T cells and monocytes, which further induce inflammatory responses [18, 25, 26]. These data suggest that loss of JKAP in T cells could contribute to dysregulation of the immune complement system and inflammatory responses in kidneys, leading to lupus nephritis.

In this study, we demonstrated that JKAP protein levels in T cells were significantly decreased in active lupus nephritis patients compared to either inactive lupus nephritis or no nephritis SLE patients. Although we only enrolled 12 patients with active SLE nephritis (8 of them received renal biopsy), we showed that lower JKAP protein levels were correlated with renal SLEDAI scores. Remarkably, the diagnostic power of JKAP downregulation is higher than that of C3/C4 reduction or anti-dsDNA enhancement. Thus, the JKAP protein level in T cells is a useful diagnostic biomarker for active lupus nephritis. Moreover, JKAP downregulation in T cells was correlated with poor renal outcome of lupus nephritis patients. The findings suggest that the JKAP protein is also a novel prognostic biomarker for lupus nephritis.

Active lupus nephritis patients are commonly treated with immunosuppressants, which may inhibit T-cell activation and induce lymphocyte death [15]. Therefore, the observation of reduced JKAP levels in these patients could be related to therapeutic agents. In this cohort, two enrolled subjects were newly diagnosed, treatment-naïve lupus patients. Their relative JKAP to actin levels were very low (JKAP/actin = 0.02 fold), indicating that low JKAP expression was related to lupus activity. Longitudinal follow-up study of JKAP expression levels from another lupus patient even showed an increased pattern (from JKAP/actin = 0.13 fold to 0.39 fold) after glucocorticoid and azathioprine therapy. Moreover, JKAP protein levels were not correlated with treatments of glucocorticoid or immunosuppressive agent (data not shown). The data indicate that JKAP protein levels are not significantly downregulated by immunosuppressive treatment.

In recent years, linkage analysis and genome-wide association studies (GWAS) have been used for analyses of genetic variants in SLE [27]. The investigation of variations at the genetic and epigenetic levels may lead to identification of useful serum and urine protein biomarkers in SLE patients. More genetic risk loci of autoimmune diseases remain to be uncovered. It is also important to examine the genetic and epigenetic regulations of JKAP in SLE, especially those with lupus nephritis, in the future.

The key finding of this study is that JKAP protein levels were significantly downregulated in peripheral blood T cells isolated from SLE patients with active lupus nephritis. Greatly decreased JKAP levels in peripheral blood T cells may contribute to T-cell hyperactivation, leading to the progression of SLE. Monitoring the JKAP protein levels in peripheral blood T cells will help to evaluate disease activity and the risk for kidney damage. Thus, determination of JKAP levels in T cells may facilitate early diagnosis and intervention for improving favorable outcomes in SLE, particular for those patients with active lupus nephritis.

## MATERIALS AND METHODS

### Study design and human subjects

Forty-three patients who were diagnosed with SLE based on the 1997 revised criteria of the American College of Rheumatology for SLE were enrolled [28]. All patients were requested to submit a written informed consent form approved by the Institutional Review Board, Taichung Veterans General Hospital, Taiwan between September 2010 and October 2015. Disease activity was assessed using the SLE Disease Activity Index (SLEDAI) [29]. Complete renal remission was defined by serum creatinine ≤ 1.4 mg/dl and proteinuria of ≤ 0.33 g/d [30]. Thirty-two healthy volunteers who had no rheumatic or renal diseases served as controls; written informed consent was also obtained from individual volunteers. All experiments were performed in accordance with the guidelines approved by the Institutional Review Board, Taichung Veterans General Hospital, Taiwan. The human CD3^+^ T cells were freshly isolated and negatively selected from the peripheral blood leukocytes of clinical samples using a cocktail of biotin-conjugated anti-CD19, anti-CD14, and anti-CD11b antibodies (Biolegend) on a MACS column (Miltenyi Biotec).

### Mice

A Myc-tagged full-length human dominant-negative JKAP mutant (JKAP-C88S) coding sequence was placed downstream of the proximal *lck* promoter [31]. The Lck proximal promoter also drives gene expression in peripheral T lymphocytes in addition to thymocytes [32]. Transgenic mice were generated by pronuclear microinjection in C57BL/6 background. The genotype of the offspring was determined by PCR analysis. The data presented in this study reflect experiments performed on sex-matched, 4-72-week-old littermates. All animal experiments were performed in accordance with guidelines and protocols approved by the Institutional Animal Care and Use Committee of the National Health Research Institutes, Taiwan.

### Antibodies and reagents

Anti-JKAP antibody (clone #3D3) purchased from Abnova, anti-phospho-Y394-Lck antibody (#2101) purchased from Cell Signaling, anti-myc antibody (#9E10) purchased from Millipore, and anti-β-actin antibody (#AC-74) purchased from Sigma-Aldrich were used for immunoblotting. GFP-tagged JKAP (WT) and JKAP C88S plasmids were reported previously [14]. The SuperSignal chemiluminescent reagent was from Thermo. The rest of the chemicals were purchased from Sigma-Aldrich.

### Immunoblotting analysis

The cell extracts were fractionated on SDS-PAGE and transferred to PVDF membranes. The membranes were probed, first with individual primary antibodies and then with horseradish peroxidase (HRP)-conjugated secondary antibody. The HRP substrate reaction was performed using the SuperSignal chemiluminescent reagent, and the chemiluminescent signal was detected by a BioSpectrum 500 imaging system. Full blots are shown in Supplementary Figure S8.

### Enzyme-linked immunosorbent assays (ELISAs)

The levels of IL-2, IL-4, IFN-γ, IL-17A, and IL-6 in mouse sera were analyzed by individual ELISA kits purchased from eBioscience. The levels of anti-nuclear antibodies (ANA/ENA) and anti-dsDNA antibodies in mouse sera were analyzed by ELISA kits purchased from Alpha Diagnostic International. The cytokines in the kidney fluids of mice were analyzed using Proteome Profiler^TM^ Array purchased from R&D. The levels of C5a, IL-16, soluble ICAM-1 (sICAM-1), and soluble VCAM-1 (sVCAM-1) in T-cell culture supernatants or mouse urines were analyzed by individual ELISA kits purchased from R&D.

### Flow cytometry analyses

Peripheral blood leukocytes were isolated from human subjects, followed by cell-surface marker staining and intracellular staining using the protocol described previously [33, 34]. The antibodies used for staining are as follows. Anti- hCD3-PE-Cy7 (SK7) and anti-hIL-17A-APC (N49-653) antibodies were purchased from BD Biosciences. Anti-CD4-pacific blue and anti-CD8-PerCP-Cy5.5 antibodies were purchased from Biolegend.

### Densitometry and statistical analysis

Densitometry analysis of the immunoblotting results was performed using GelPro software (Media Cybernetics). The adequate sample size was determined by the power calculations using G*Power 3.1.6 software. Achieved powers at individual animal experiments were all higher than 0.9. The *Mann*-Whitney *U test* or Kruskal-Wallis test was used for statistical analyses. The correlations between JKAP protein levels and clinical manifestations were evaluated using Spearman's rank correlation coefficient (*r*). Bonferoni's correction for multiple comparisons was analyzed by post-hoc analysis. Complete renal remission pattern was analyzed by Kaplan-Meier method. *P* value of less than 0.05 was considered statistically significant. All statistical analyses of clinical data were independently verified by two senior biostatisticians at Taichung Veterans General Hospital.

## SUPPLEMENTARY MATERIAL FIGURES AND TABLE


