# Supplementary material for: Downregulation of the phosphatase JKAP/DUSP22 in T cells as a potential new biomarker of systemic lupus erythematosus nephritis
Source: Oncotarget. 2016 Aug 19;7(36):57593–605. doi: 10.18632/oncotarget.11419 (PMC5295375; doi:10.18632/oncotarget.11419)
Supplement: Supplementary file 1 [file oncotarget-07-57593-s001.pdf]

Downregulation of the phosphatase JKAP/DUSP22 in T cells as a potential new biomarker of systemic lupus erythematosus nephritis

Supplementary Material

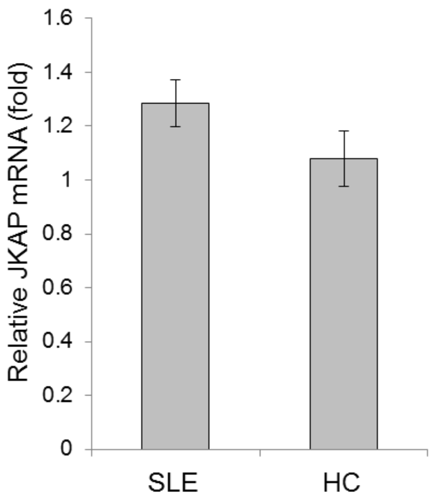

Supplementary Figure S1: JKAP mRNA levels in T cells are similar between SLE patients and healthy controls. JKAP mRNA levels in peripheral blood T cells from individual subjects (14 SLE patients and 12 healthy controls) were analyzed by real-time PCR. Data are expressed as the ratio of JKAP to peptidylprolyl isomerase A (PPIA) mRNA transcripts. Bars show the mean ± SEM.

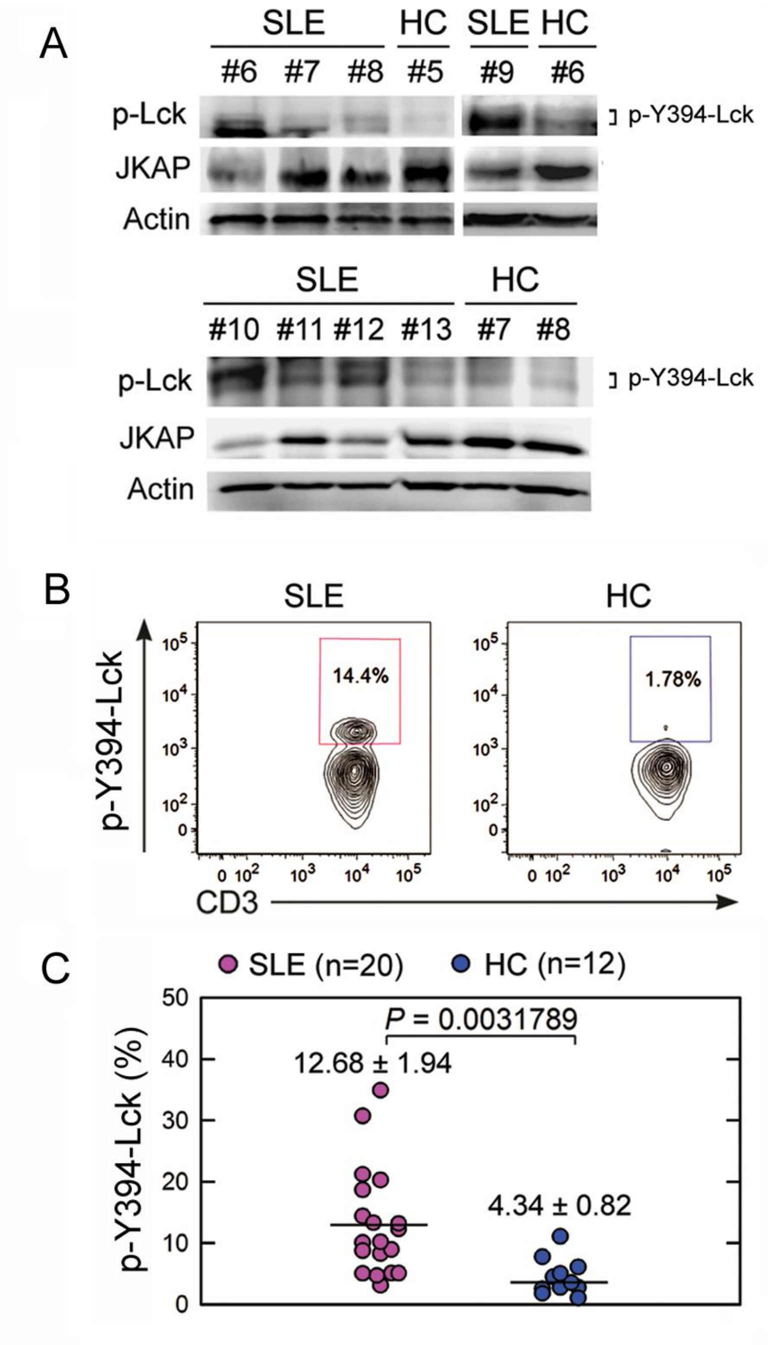

Supplementary Figure S2: Lck phosphorylation is enhanced in T cells from human SLE patients. A. Immunoblotting of phospho-Y394-Lck (p-Lck) and JKAP levels in purified peripheral blood T cells from eight SLE patients and four healthy controls (HC). **B.** Flow cytometry analyses of phospho-Y394-Lck-positive T cells in peripheral blood from 20 SLE patients and 12 healthy controls (HC); results from one SLE patient and one healthy control are shown as representative data. **C.** Individual percentages of phospho-Y394-Lck-positive T cells described in panel B are shown. Achieved power = 1.0. Two-tailed Student's *t*-test, *P* = 0.0031789.

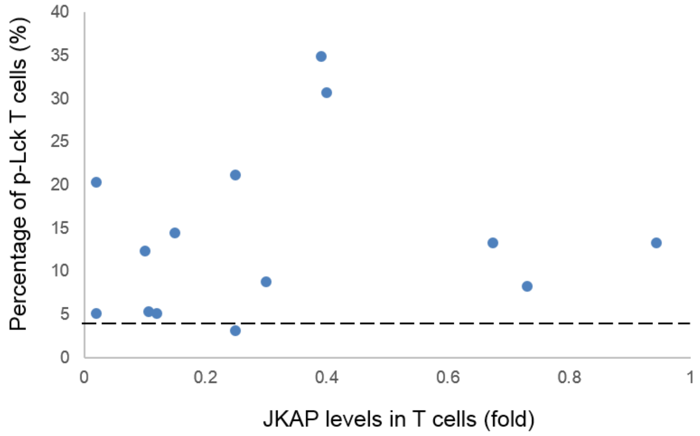

Supplementary Figure S3: SLE patients with normal JKAP levels in T cells also had increased population of phospho-Lck-positive T cells. Individual JKAP levels of T cells and percentages of phospho-Lck-positive (p-Lck) T cells from 14 SLE patients. A dotted line denotes the average percentage of p-Lck T cells in peripheral blood leukocytes of healthy controls.

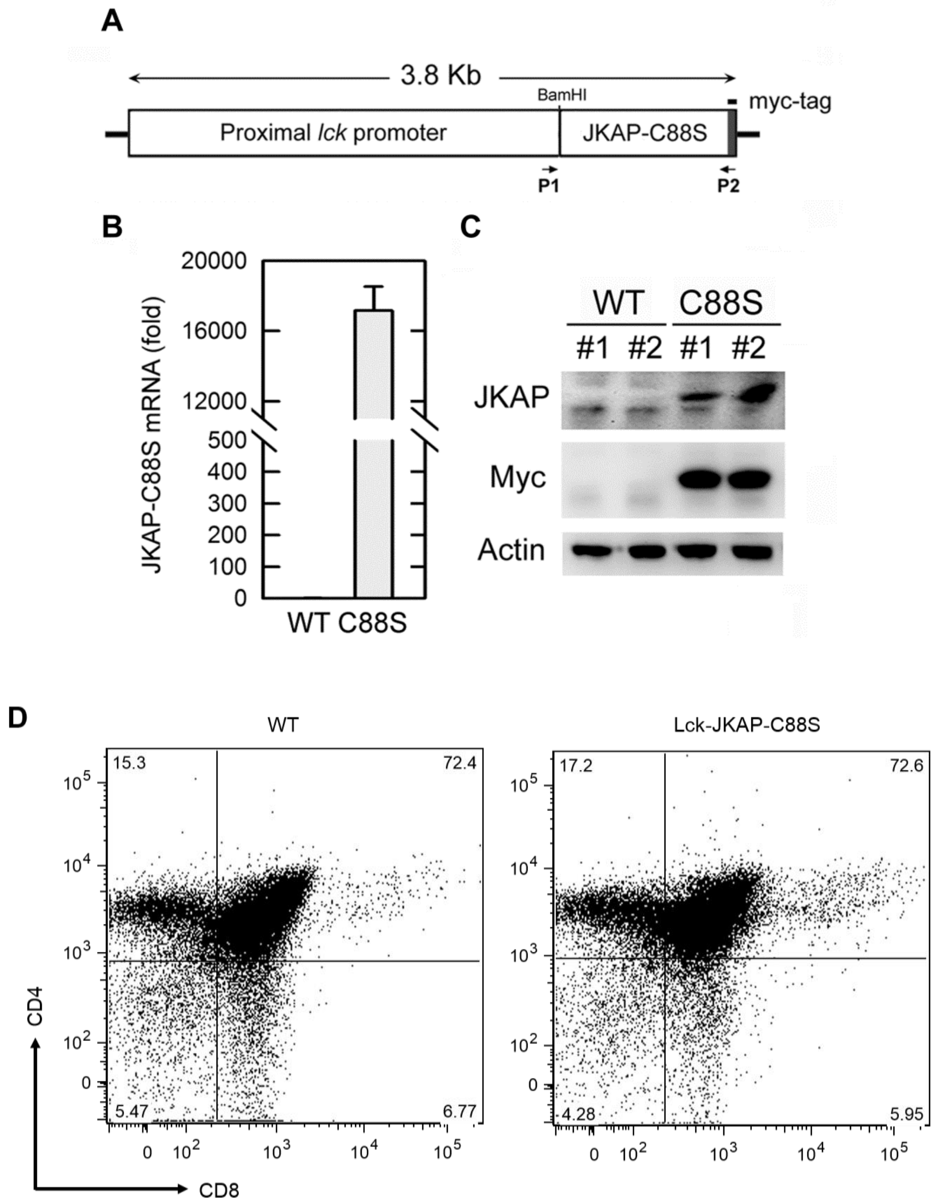

Supplementary Figure S4: Generation of the Lck-JKAP-C88S transgenic mouse. A. The schematic diagram of the Lck-JKAP-C88S construction. **B.** Real-time PCR analyses of transgenic human JKAP-C88S mRNA levels in murine splenic T cells. Relative fold changes were normalized to GAPDH mRNA levels. **C.** Immunoblotting of transgenic human Myc-tagged JKAP-C88S proteins in murine splenic T cells. **D.** T-cell lineages in the thymus of wild-type and Lck-JKAP-C88S transgenic mice. WT, wild-type mice; C88S, Lck-JKAP-C88S transgenic mice.

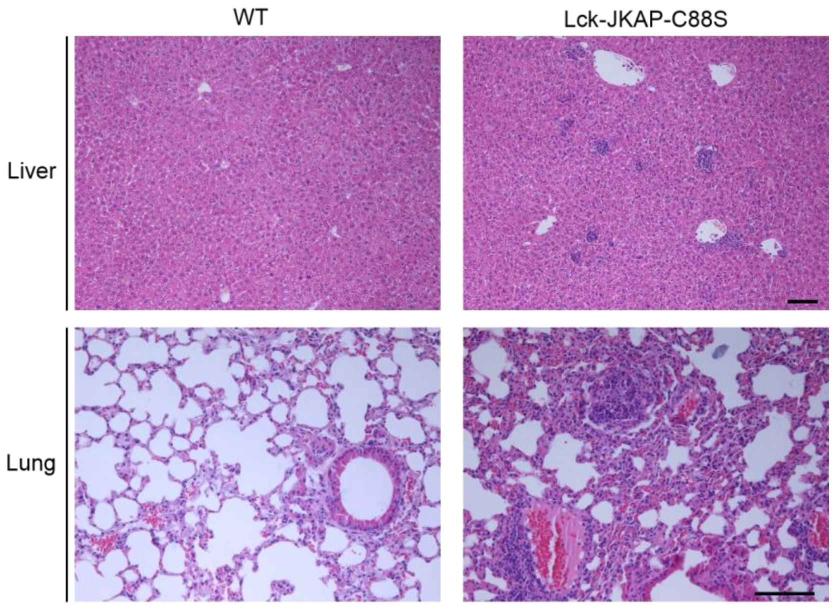

Supplementary Figure S5: Lck-JKAP-C88S transgenic mice develop spontaneous inflammation in the liver and the lungs. Photomicrographs of the liver and lung tissues from 24-week-old Lck-JKAP-C88S transgenic mice and age-matched wild-type mice stained with hematoxylin-eosin. Scale bar: 100 μm. WT, wild-type mice.

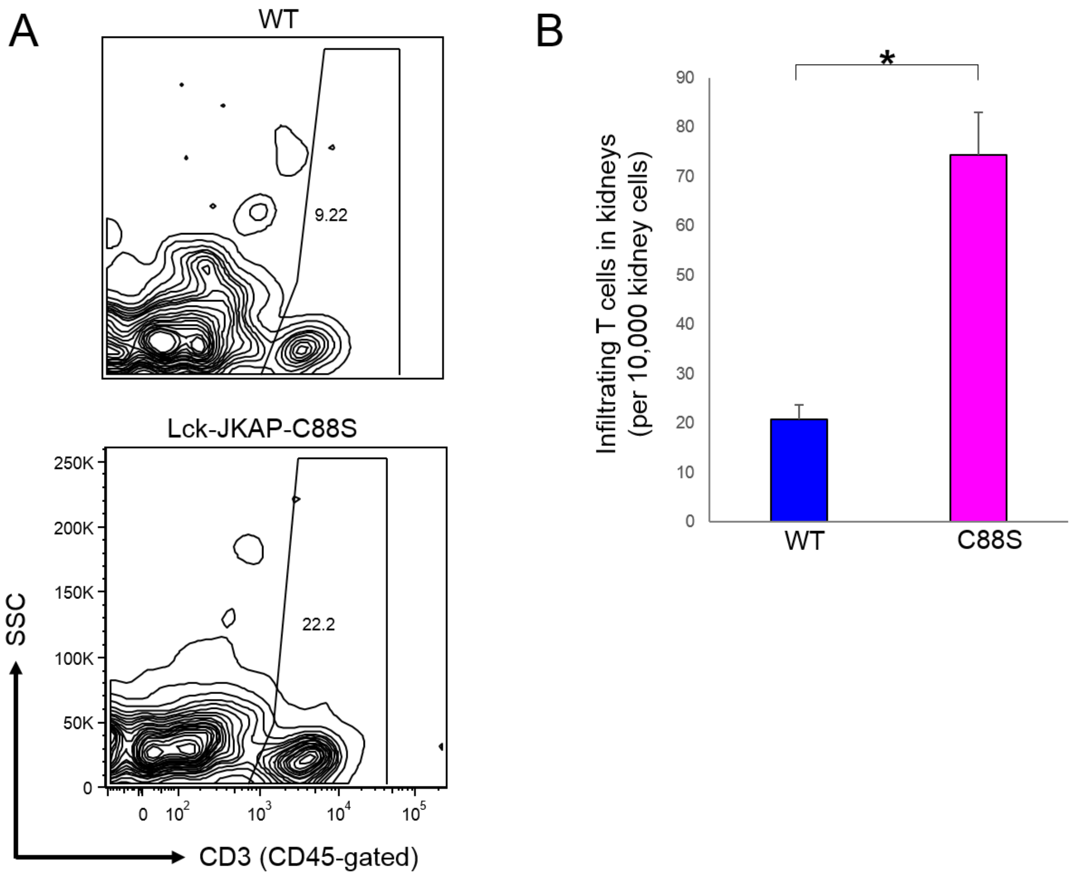

Supplementary Figure S6: The population of infiltrating T cells in the kidneys is enhanced in Lck-JKAP-C88S transgenic mice. Cells were isolated from the kidneys of 24-week-old WT or Lck-JKAP-C88S transgenic mice. **A.** Cells were stained for expression of CD45 or CD3, and then cells were analyzed by flow cytometry. The contour plots show percentages of infiltrating $CD3^+$ T cells among the $CD45^+$ leukocytes in the kidneys of mice. **B.** The numbers of infiltrating T cells are normalized to 10,000 cells in the kidneys. Two-tailed Student's *t*-test, *, $P < 0.05$. Bars show the mean ± SEM. WT, wild-type mice; C88S, Lck-JKAP-C88S transgenic mice.

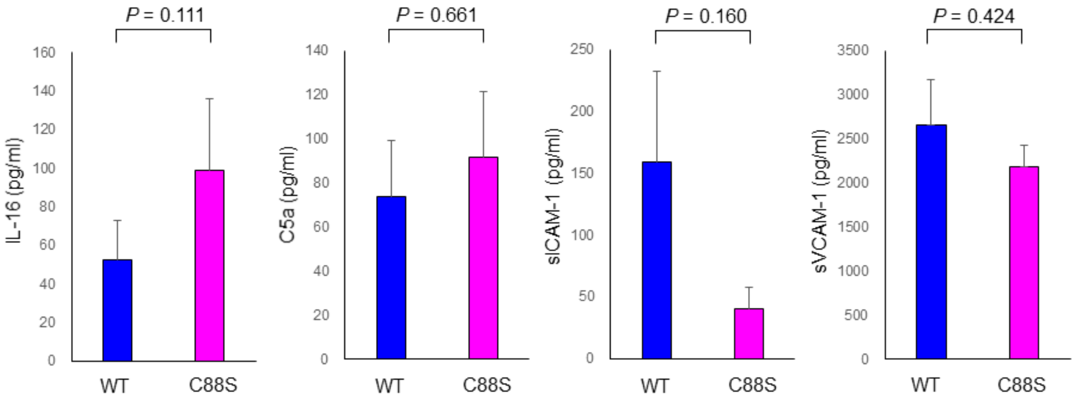

Supplementary Figure S7: The levels of complement component C5a and cytokines are not significantly increased in the urine of Lck-JKAP-C88S transgenic mice. Twenty-four-hour urine from 6-month-old wild-type and Lck-JKAP-C88S transgenic mice were subjected to ELISA assays for IL-16, C5a, soluble ICAM-1 (sICAM-1), and soluble VCAM-1 (sVCAM-1). *P* values were calculated with the two-tailed Student's *t*-test. Bars show the mean ± SEM. WT, wild-type mice; C88S, Lck-JKAP-C88S transgenic mice.

Figure 1A

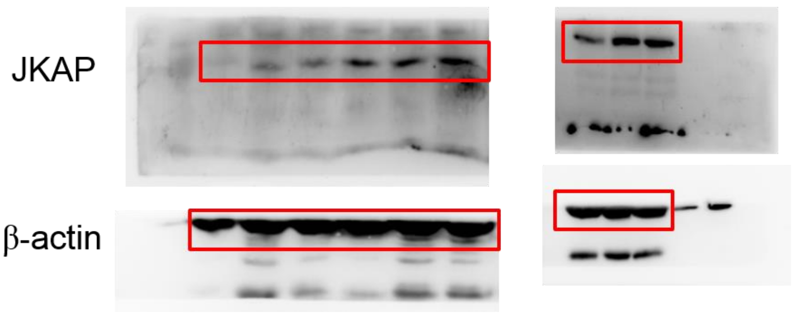

Supplementary Figure S8: Full immunoblots with indicated areas of selection.

Supplementary Table S1: Comparison of phospho-Y394-Lck-positive T cells with various symptoms among SLE patients.

Symptom	n	Phospho-Lck T cells (%)	*P*
Fever[†]			
Yes	4	16.33 ± 14.31	1.000
No	10	13.07 ± 8.02	
Rash[†]			
Yes	9	14.16 ± 9.13	0.739
No	5	13.73 ± 11.79	
Arthritis[†]			
Yes	9	12.63 ± 8.83	0.505
No	5	16.47 ± 11.76	
Cutaneous vasculitis[†]			
Yes	4	15.78 ± 13.31	0.777
No	10	13.29 ± 8.66	
Oral ulcer[†]			
Yes	2	9.95 ± 6.58	0.715
No	12	14.71 ± 10.17	
Serositis[†]			
Yes	4	20.37 ± 12.20	0.203
No	10	11.46 ± 7.83	
CNS[†]			
Yes	1	5.06	0.172
No	13	14.69 ± 9.73	
Nephritis[¶]			
Active	8	18.15 ± 10.10	0.089
Inactive	4	6.31 ± 2.68	
No	2	12.83 ± 10.56	

Plus-minus values are means ± SD.

[†] *P* values were calculated with the *Man*n–Whitney *U* test.

[¶] *P* values were calculated with the Kruskal–Wallis test.

P-value < 0.05, statistical significance.
